# Effects of pregnancy and lactation-associated osteoporosis on children: analysis using linked mother and child data

**DOI:** 10.1007/s00774-026-01720-4

**Published:** 2026-04-11

**Authors:** Kyoko Kasahara, Shunichiro Tsuji, Taku Kawasaki, Takashi Murakami

**Affiliations:** 1 Department of Obstetrics and Gynecology, Shiga University of Medical Science, Seta Tsukinowa-Cho, Otsu, Shiga 520-2192 Japan; 2 Department of Orthopedic Surgery, Shiga University of Medical Science, Seta Tsukinowa-Cho, Otsu, Shiga 520-2192 Japan

**Keywords:** Children, Fracture, Lactation, Osteoporosis, Pregnancy

## Abstract

**Introduction:**

The health status of children born to mothers with pregnancy and lactation-associated osteoporosis (PLO) and impaired bone homeostasis remains unknown. This study aimed to investigate the effects of maternal PLO on child health, the PLO-predisposing factors related to such effects, and the incidence of the condition.

**Materials and methods:**

From a claims dataset comprising 172,730 mothers linked with their 210,124 children, mothers who developed fragility fractures between 5 months before and 12 months after delivery, along with their children, were defined as the PLO group. Healthy controls were randomly selected by matching maternal age and child sex. Body mass index (BMI), drug use, and maternal and pediatric diseases were compared between the two groups.

**Results:**

Fifty-nine mothers and 65 children were included in the PLO group, and 11,556 mothers and 11,664 children served as the control group. The most common PLO fracture was a vertebral fracture, and delayed diagnosis was common. Maternal PLO had few adverse effects on child health. There were trends toward a higher frequency of birth asphyxia in singletons (*P* = 0.072) and low birth weight in twins (*P* = 0.070) in the case children, but with no significant differences. The incidence of PLO was estimated at 374 per million deliveries. Multiple delivery, maternal age ≥ 40 years, low BMI, heparin use, and ovulation disorder were identified as predisposing factors for PLO.

**Conclusion:**

There is little evidence that maternal PLO adversely affects child health. The predisposing factors for PLO suggest that caution is warranted regarding current advancements in reproductive technology.

**Supplementary Information:**

The online version contains supplementary material available at 10.1007/s00774-026-01720-4.

## Introduction

Pregnancy and lactation-associated osteoporosis (PLO) is a major type of premenopausal osteoporosis in which pregnant and lactating women present with fragility fractures, primarily in the vertebrae [1, 2]. Although our knowledge of the pathophysiology of this rare condition remains limited, various observational studies on PLO have been conducted. The high prevalence of osteoporosis or osteoporotic fracture among second-degree relatives of women with PLO [3–5] suggests that hereditary factors are involved in its etiology. These epidemiological findings are supported by recent studies identifying the pathogenic variants in bone-specific genes in PLO patients [6, 7].

In addition, the observation that ovulatory disorders [2], low body mass index (BMI) (< 18 kg/m^2^) [8], and advanced maternal age [2, 9–11] are related to the development of PLO is not unexpected, as low estrogen levels and aging are established risk factors for low bone mass. Treatment with drugs predisposing to osteoporosis, such as corticosteroids, heparin, and antiseizure medications, is also linked to the occurrence of PLO [8, 9]. Not only do these factors and drugs indicate a strong association with maternal conditions, but the well-known familial effect [3–7] also suggests that maternal PLO might affect child health. Nevertheless, the health status of children born to mothers with PLO has been investigated in only a few case reports [12].

In general, pregnancy and lactation are well-known conditions involving high bone turnover to provide infants with sufficient calcium for growth [13, 14]. Specifically, lactation induces bone mass loss through maternal hormonal changes, independent of their calcium intake [15]. There is no firm evidence that low maternal calcium intake leads to impaired breast milk quality [16] or increased bone loss in healthy women [17, 18]. Thus, while maternal physiological changes appear to ensure an adequate calcium supply, the positive correlation between low gestational weight gain and low birthweight or prematurity [19–21] might indicate that women in negative calcium balance may fail to provide sufficient calcium. Furthermore, increasing evidence suggests decreased bone turnover in PLO patients, which is contrary to the states of normal pregnancy and lactation [6, 7, 22, 23].

The primary aim of this study is to investigate the effects of maternal PLO on the health status of their children. We hypothesized that children born to mothers with PLO may have impaired health because the calcium supply from mothers with imbalanced bone turnover fails to meet the infant’s needs. Although we previously studied the epidemiology of PLO and reported its incidence using a claims database consisting of only parous women [2], this study utilizes a new dataset in which mother and child data are linked [24]. This approach is more useful for investigating the long-term effects of a rare maternal condition on children. Retrospective studies based on individual medical records are often insufficient for investigating such conditions because mothers and their children usually see different clinicians, and patients often visit multiple hospitals throughout their lives. Furthermore, we considered the new dataset [24] to be superior to the previous one with no child data [2] for studying the epidemiology of this condition, which is strongly related to reproduction and parenting. The second aim is to study the factors predisposing to PLO, which may contribute to its effects on children. The third aim is to re-estimate the incidence of PLO using the new dataset.

## Materials and methods

### Data source

A cohort study was conducted to clarify the epidemiological features of PLO, and a nested case–control study was conducted to compare PLO cases and controls using an administrative claims database managed by the Japan Medical Data Center (JMDC, Tokyo, Japan). This database contains health insurance claims, annual health check-up data, and ledger data from employee-based insurance plans in Japan. Disease diagnoses, procedures, and drugs are recorded using ICD-10 codes, Japanese standardized procedure codes, and the Anatomical Therapeutic Chemical (ATC) Classification of both the European Pharmaceutical Market Research Association (EphMRA) and the World Health Organization (WHO) (Table S1). Codes assigned to inpatients are distinguishable from those assigned to outpatients by insurance type. To protect anonymity, the exact date of birth is not provided, but is recorded on a monthly basis.

From the JMDC database, records of 172,730 mothers and 210,124 children recorded from January 2005 to December 2021 were extracted. The mothers and children met the following criteria: 1) the child was born between January 2006 and December 2020, the date of birth and insurance enrollment date were identical, and the eligible mother was found in the same family; and 2) the mother was the insured person or spouse in the same family as the child at the time of the child’s birth, had an ICD-10 code for an obstetrical condition (O00-O99), and her observation started one year or more before the child’s birth.

This study was conducted in accordance with the Declaration of Helsinki and was approved by the University Research Ethics Board (No. R2024-027), including the use of an administrative claims database without need for individual consent.

### Study cohort

The study cohort included the mothers linked to their children using the family identification code, with no history of insurance withdrawal and with data available one year or more after the child’s birth, and their children. The child’s date of birth was considered the mother’s date of delivery. Two or more children born at the same time who were linked to one mother were considered multiple births.

### Identification of PLO cases

As in our prior study [2], PLO fractures were defined as fragility fractures of vertebrae, pubis, pelvis excluding the pubis, and proximal femur that were diagnosed between 5 months before and 12 months after delivery (from -5M to + 12M after delivery, with the month of delivery as 0M). Old proximal femoral fractures were excluded. Disease diagnoses flagged for suspicion were also excluded.

The procedure for identifying mothers with PLO was as follows. First, mothers with at least one ICD-10 code for PLO fractures from -5M to + 12M after delivery were extracted. Subsequently, fracture records were examined in detail to identify low-trauma fractures. Multiple fractures involving multiple sites, transverse process fractures, dislocated fractures, and coincident severe traumatic conditions were excluded as high-trauma fractures, though multiple vertebral fractures were not excluded. Osteogenesis imperfecta and secondary malignant bone neoplasms were also excluded. Mothers who developed a fracture outside the age range of 18–47 years were excluded to avoid including fractures occurring during the growth or postmenopausal period. Furthermore, mothers with neither imaging examinations nor treatment (including prescriptions of analgesics and back braces) in the same month as the fractures were excluded, in a departure from our prior study [2], to ensure only confirmed fracture patients were included. Therefore, Japanese standardized procedure codes for imaging examinations and back braces, as well as the ATC codes for oral analgesics and cold patches with analgesics, were investigated (Table S1). In addition, children born unrelated to the mother’s fracture were excluded. Finally, mothers who developed at least one PLO fracture and their “fracture-related” children were defined as the PLO group, and they were divided into single delivery and multiple delivery groups.

### Control selection

The control mothers and their children were selected from the study cohort. First, mothers without any ICD-10 code for fragility fractures involving vertebrae, proximal femur, pubis, pelvis excluding the pubis, sacrum, proximal humerus, and distal radius, which are common fragility fractures in the elderly [2], and their children were extracted. Of these, the minimum number of candidates per PLO mother was 221 for the single delivery group and 18 for the multiple delivery group. Therefore, 216 controls per one PLO mother were randomly selected by matching maternal age and sex of the children for the single delivery group, whereas 18 controls per one PLO mother were randomly selected for the multiple delivery group. This resulted in a matching ratio 12 times higher for the single delivery group than for the multiple delivery group. Children with a shorter observation period than those born to PLO mothers were not selected. In addition, children with a long observation period were given a higher chance of being selected.

### Definitions of variables

#### Musculoskeletal complaints requiring treatment

In the PLO mothers, the ICD-10 codes for the diseases that could cause musculoskeletal complaints requiring treatment before their fracture diagnosis were investigated (Table S1), suspecting that such complaints might mean real fracture onset. Regarding such treatment, the Japanese standardized procedure codes for back braces and the ATC codes for analgesics were investigated. The PLO mothers who were assigned both codes for these diseases and treatment in the same month before their fracture diagnosis were suspected delayed-diagnosis cases.

#### Body mass index

BMI values were obtained from annual health check-up data. Defining the pregnancy period as -8M to 0M after delivery, the pre-pregnancy and post-pregnancy periods were set as 36 months before and after the pregnancy period, respectively. BMI was defined as the latest BMI value in the pre- pregnancy period or the earliest BMI value in the post- pregnancy period, with the former given priority.

#### Drug use and diseases related to maternal fracture risk

As drugs given to mothers, the ATC codes of anticoagulants such as unfractionated heparin (UFH) and warfarin, glucocorticoids (GCs), and five kinds of oral antiseizure medications (ASMs) related to osteoporosis in the practice guidelines for epilepsy in Japan, i.e., phenobarbital, primidone, phenytoin, carbamazepine, and valproic acid, were investigated.

UFH use was defined as receiving more than 56 ampoules of UFH during two consecutive months before or including the same month of delivery. Warfarin use was defined as receiving warfarin for more than 28 prescription days during two consecutive months prior to five months before delivery. GC use was defined as GC injections with more than three treatment days during two consecutive months or oral GCs for more than 28 prescription days during two consecutive months prior to five months before delivery. ASM use was defined as at least one of five kinds of ASMs for more than 28 days during two consecutive months prior to five months before delivery.

As diseases of mothers, ICD-10 codes for idiopathic thrombocytopenic purpura, thyroid gland disorders, diabetes mellitus, eating disorders, epilepsy, rheumatoid arthritis (RA), systemic lupus erythematosus, dermatopolymyositis, “absent, scanty, and rare menstruation”, “female infertility associated with anovulation”, and other types of female infertility were investigated (Table S1). Respiratory diseases and skin diseases that often require GCs were also investigated. A woman who was assigned an ICD-code for these diseases prior to five months before delivery was defined as a patient with that disease. When aggregating, “Absent, scanty, and rare menstruation” and “female infertility associated with anovulation” were combined as “ovulation disorder”, whereas other types of female infertility were combined as “female infertility due to other than ovulation disorder”. The disease diagnoses flagged for suspicion were excluded.

#### Diseases of children

The ICD-10 codes for neonatal diseases such as preterm infant, low birth weight, birth asphyxia, hypothermia, hypoglycemia, respiratory disorders, and jaundice were investigated. The standard disease code for hyperbilirubinemia was investigated as neonatal jaundice. A child assigned a code of a disease as an inpatient in the same or the next month of birth was considered a patient with that disease. The disease diagnoses flagged for suspicion were excluded.

In addition, the codes for malnutrition, including emaciation, vitamin D deficiency (rickets), obesity, disorders of psychological development, lack of expected normal physiological development, fractures, and infectious diseases were investigated as diseases up to childhood. A child assigned a code of an infectious disease as an inpatient was defined as having a “hospitalization due to infectious diseases”, whereas a child assigned a code of a disease other than an infectious disease was defined as having that disease regardless of admission. The disease diagnoses flagged for suspicion were excluded.

### Statistical analysis

The incidence of PLO in the study cohort was estimated using Poisson regression. The associations of PLO risk, multiple deliveries, and maternal age were also assessed by Poisson regression. Case–control comparisons of BMI and underweight of the mothers were performed by the Mann–Whitney *U* test and Fisher’s exact test, respectively. Case–control comparisons of drug use and diseases related to maternal fracture risk were quantified by odds ratios, and hypothesis testing was done by Fisher’s exact test. The association between the risk of perinatal disorders of the children and PLO of the mothers was assessed by risk ratios, and hypothesis testing was done using Fisher’s exact test. The associations between the risk of other disorders of the children and PLO of the mothers were evaluated in a time-to-event fashion via Cox regression analysis and the log-rank test.

All statistical analyses were performed using SAS 9.4 (SAS Institute Inc., Cary, NC, USA). A two-sided significance level of 5% was used for all tests.

## Results

### Identification of mothers with PLO fractures and their children

**Fig. 1:**
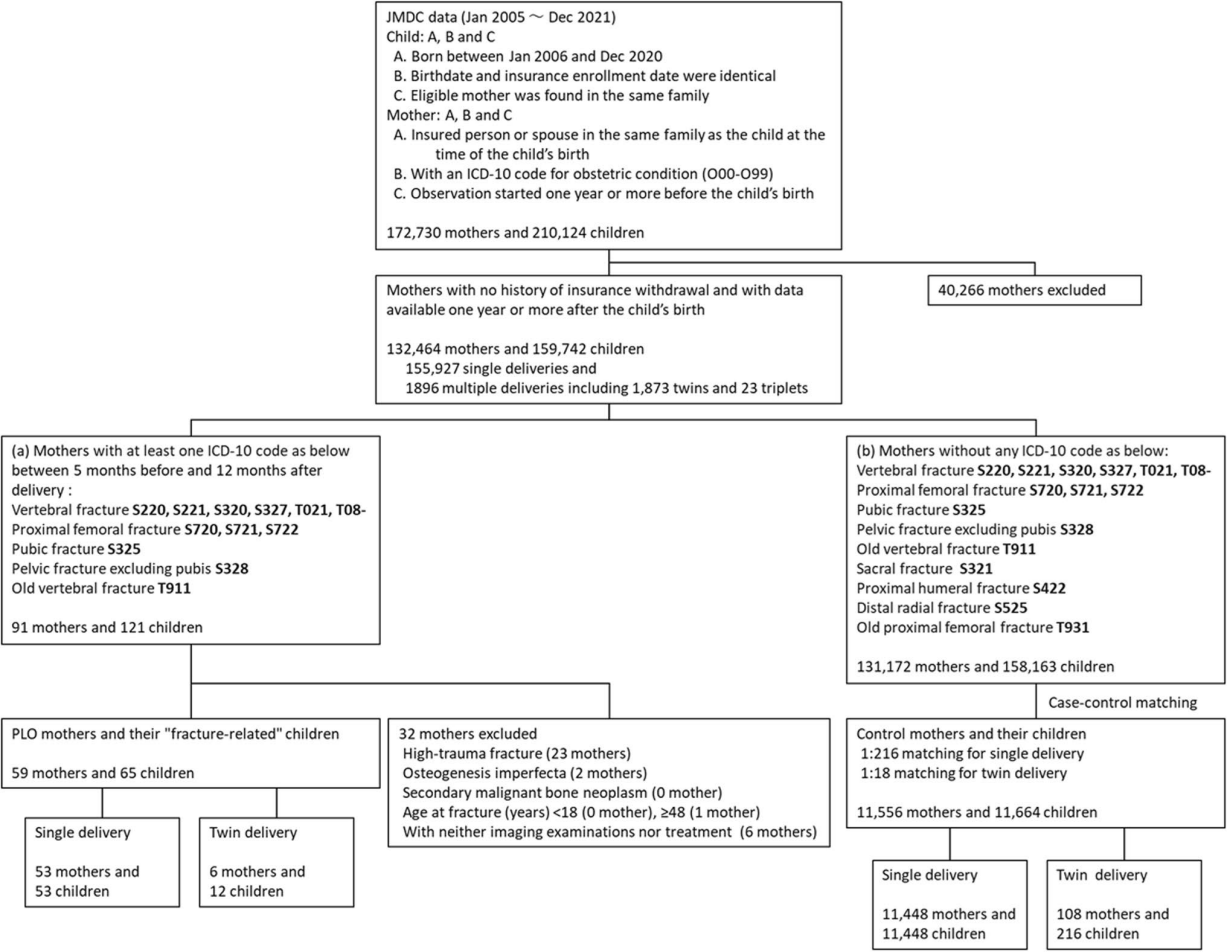
Flowchart of the study population. Of the172,730 mothers who met the inclusion criteria, two groups were identified: **a** 91 mothers with at least one ICD code for a PLO fracture, and **b** 131,172 mothers without any ICD-10 codes for common fragility fractures seen in the elderly. Some mothers were included in neither of these groups. From group (a), 59 mothers with PLO fractures (aged 18–47 years) and their 65 “fracture-related” children were defined as the PLO group, which comprised 53 mother–child pairs from single deliveries and 6 mother–child pairs from twin deliveries. In contrast, the control group was selected from group (b). Random matching by maternal age and child sex yielded 11,448 control mother–child pairs for single deliveries and 108 control mother–child pairs for twin deliveries. JMDC, Japan Medical Data Center; ICD-10, International Classification of Diseases, 10th revision; PLO, pregnancy and lactation-associated osteoporosis

A total of 132,464 mothers had no history of insurance withdrawal and had data for one year or more after their child’s birth (Fig. 1). Of these mothers, 91 had at least one ICD code for a PLO fracture. Among these 91 mothers, those with high-trauma fractures (*n* = 23), osteogenesis imperfecta (*n* = 2), advanced age at the time of fracture (n = 1; 61 years old), or a lack of imaging examinations and treatment at the time of the fracture (*n* = 6) were excluded. No mothers had secondary malignant bone neoplasms, and none developed such fractures at the age of 17 years or younger. Therefore, 59 mothers between the ages of 18 and 47 years were identified as having PLO fractures. No mother developed a PLO fracture more than once. The PLO group consisted of 53 mother–child pairs from single deliveries and 6 pairs from twin deliveries (6 mothers and 12 children). No mother had a triplet delivery.

### Characteristics of mothers with pregnancy and lactation-associated osteoporosis

Among the 59 mothers with PLO, the median maternal age at the time of single and twin deliveries was 35 and 31 years, respectively (Table S2). The most common fracture site was the vertebrae (49/59, 83.1%), followed by the pubis (8/59, 13.6%), pelvis excluding the pubis (1/59, 1.7%), and proximal femur (1/59, 1.7%).

The diagnosis of PLO fractures was most frequent at two months postpartum and decreased gradually until approximately one year after delivery (Fig. S1a). In total, 48 of the 59 (81.4%) PLO fractures developed after the month of delivery. PLO fractures in mothers with twins were concentrated near the time of delivery. Although not presented in the figure, more than half of the pubic fractures (5/8) and all pelvic fractures excluding the pubis (1/1) were diagnosed between -1M and + 1M. The single proximal femoral fracture was diagnosed at + 11M.

Of the 59 PLO mothers, 27 (45.8%) had both codes for the diseases potentially causing musculoskeletal complaints and had received treatment for these diseases before their fracture diagnosis. The temporal distribution of PLO fracture diagnosis, excluding these 27 cases, is shown in Fig. S1b. The diagnosis of these 32 PLO fractures peaked at the month of delivery and decreased gradually until about one year after delivery. In contrast, the remaining 27 mothers were presumed to have had musculoskeletal complaints requiring treatment between one and eight months before their fracture diagnosis (Table S3). Thirteen of these 27 (48.1%) had such complaints in the month of delivery.

### Incidence of pregnancy and lactation-associated osteoporosis

The study cohort (132,464 mothers and 159,742 children) comprised 155,927 single deliveries and 1,896 multiple deliveries (including 1,873 twins and 23 triplets), totaling 157,823 deliveries. The incidence of PLO was estimated at 374 per million deliveries (Table 1). Furthermore, the incidence of PLO for single and multiple deliveries was estimated at 340 and 3,165 per million deliveries, respectively, indicating that the incidence is significantly higher in multiple deliveries than in single deliveries (RR = 9.3; 95% CI: 4.0, 21.7; *P* < 0.001).

**Table 1:** Incidence of PLO in the study cohort

Cohort	Deliveries, *n*	PLO, *n* (%)	PLO incidence^a^ [95% CI]	Risk ratio [95% CI]	*P *value^b^
Full cohort	157,823	59 (0.037)	374 [290, 483]		
Single delivery	155,927	53 (0.034)	340 [260, 445]	Reference	
Multiple delivery	1,896	6 (0.316)	3,165 [1,422, 7,044]	9.3 [4.0, 21.7]	< 0.001

CI, confidence interval; PLO, pregnancy and lactation-associated osteoporosis

^a^PLO incidence per 1,000,000 deliveries

^b^*P* value for Poisson regression

Next, considering that PLO is typically regarded as a fragility fracture developing during the third trimester of pregnancy or early postpartum, the incidence of PLO—when defined as fractures occurring from -3M to + 2M after delivery—was 177 per million deliveries (Table S4). This incidence was also significantly greater in multiple deliveries than in single deliveries (RR = 22.4; 95% CI: 9.1, 55.3; *P* < 0.001).

Furthermore, the incidence of PLO was investigated by maternal age group, under 30 years, 30–39 years, and 40 years or older (Table 2). A maternal age of 40 years or older was positively associated with the incidence of PLO in single deliveries (RR = 3.1; 95% CI: 1.3, 7.4; *P* = 0.010).

**Table 2:** Association between maternal age and the incidence of PLO

Maternal age (y)	Deliveries, *n*	PLO, *n* (%)	PLO incidence^a^ [95% CI]	Risk ratio [95% CI]	*P* value^b^
Single delivery					
< 30	41,974	12 (0.029)	286 [162, 503]	Reference	
30–39	103,888	32 (0.031)	308 [218, 436]	1.1 [0.6, 2.1]	0.826
40–47	10,065	9 (0.089)	894 [465, 1,719]	3.1 [1.3, 7.4]	0.010
Multiple delivery					
< 30	430	2 (0.465)	4,651 [1,163, 18,597]	Reference	
30–39	1,303	4 (0.307)	3,070 [1,152, 8,179]	0.7 [0.1, 3.6]	0.631
40–47	163	0 (0)	NE		

CI, confidence interval; NE, not possible to estimate; PLO, pregnancy and lactation-associated osteoporosis

^a^PLO incidence per 1,000,000 deliveries

^b^*P* value for Poisson regression

### Comparison of the mothers in the PLO and control groups

From the 132,464 mothers with no history of insurance withdrawal and available data for at least one year postpartum, 131,172 mothers without any ICD-10 code for fragility fractures common in the elderly were extracted, along with their 158,163 children (Fig. 1). Before random selection for case–control matching, children with observation periods shorter than 15 months and their mothers were excluded, as the shortest observation period among the case children was 15 months. Subsequently, matching by maternal age and child sex yielded 11,448 control mother–child pairs for single deliveries and 108 control mother–child pairs for twin deliveries (108 mothers and 216 children).

A comparison of the characteristics of mothers in the PLO and control groups is shown in Table S5. Observation periods were significantly longer in the controls than in the PLO mothers, or were comparable between the two groups.

A comparison of the BMI values between the two study groups is shown in Table 3. In the single delivery group, BMI values were significantly lower in PLO mothers than in the control group (*P* = 0.024). There was also a trend toward a higher prevalence of underweight (BMI < 18.5 kg/m^2^) in PLO mothers than in controls, although the difference was not significant. In contrast, in the twin delivery group, no significant differences were observed in either BMI values or the prevalence of underweight between the two groups.

**Table 3:** Comparison of BMI between mothers in the PLO and control groups

	Mothers with PLO	Control	*P* value^a^
Single delivery			
Mothers having BMI records,* n* (%)	29 (54.7)	5,266 (46.0)	0.216
BMI, kg/m^2^, median (range)	18.9 (16.6, 42.6)	20.3 (13.2, 45.8)	0.024
Underweight (BMI < 18.5 kg/m^2^), *n* (%)	10 (34.5)	1,109 (21.1)	0.106
Twin delivery			
Mothers having BMI records, *n* (%)	2 (33.3)	38 (35.2)	1.000
BMI, kg/m^2^, median (range)	19.0 (18.5, 19.4)	20.2 (17.4, 33.8)	0.352
Underweight (BMI < 18.5 kg/m^2^), *n* (%)	0 (0.0)	8 (21.1)	1.000

BMI, body mass index; PLO, pregnancy and lactation-associated osteoporosis

^a^Mann-Whitney *U* test and Fisher’s exact test for continuous and categorical variables, respectively

A comparison of drug use and diseases related to maternal fracture risk between the two groups is shown in Table 4. Compared with the control group, the use of UFH, as well as the presence of ovulation disorders and RA in the single delivery group was significantly more frequent in mothers with PLO (OR = 12.1, 95% CI: 1.4, 49.1, *P* = 0.014; OR = 2.0, 95% CI: 1.1, 3.6, *P* = 0.020; and OR = 6.0, 95% CI: 0.7, 23.7, *P* = 0.048). No significant differences in the frequencies of other drugs or diseases were found between the two groups.

**Table 4:** Maternal drug use and diseases related to fracture risk: A comparison between PLO and control groups

	Mothers with PLO, *n* (%)	Control, *n* (%)	Odds ratio [95% CI]	*P* value^a^
Drug				
Glucocorticoids				
Single delivery	3 (5.7)	606 (5.3)	1.1 [0.2, 3.3]	0.759
Twin delivery	1 (16.7)	7 (6.5)	2.9 [0.1, 31.4]	0.360
Antiseizure medications				
Single delivery	0 (0.0)	53 (0.5)	0.0 [0.0, 12.8]	1.000
Twin delivery	0 (0.0)	0 (0.0)	NE	NE
Warfarin				
Single delivery	0 (0.0)	2 (0.0)	0.0 [0.0, 755.5]	1.000
Twin delivery	0 (0.0)	0 (0.0)	NE	NE
Unfractionated heparin				
Single delivery	2 (3.8)	37 (0.3)	12.1 [1.4, 49.1]	0.014
Twin delivery	0 (0.0)	1 (0.9)	0.0 [0.0, 342.0]	1.000
Disease				
Idiopathic thrombocytopenic purpura				
Single delivery	0 (0.0)	6 (0.1)	0.0 [0.0, 142.5]	1.000
Twin delivery	0 (0.0)	0 (0.0)	NE	
Thyroid gland disorders				
Single delivery	7 (13.2)	968 (8.5)	1.6 [0.6, 3.7]	0.212
Twin delivery	1 (16.7)	20 (18.5)	0.9 [0.0, 8.5]	1.000
Diabetes mellitus				
Single delivery	3 (5.7)	356 (3.1)	1.9 [0.4, 5.8]	0.229
Twin delivery	1 (16.7)	9 (8.3)	2.2 [0.0, 22.9]	0.431
Eating disorder				
Single delivery	0 (0.0)	30 (0.3)	0.0 [0.0, 23.2]	1.000
Twin delivery	0 (0.0)	0 (0.0)	NE	
Epilepsy				
Single delivery	0 (0.0)	85 (0.7)	0.0 [0.0, 7.9]	1.000
Twin delivery	0 (0.0)	0 (0.0)	NE	
Respiratory diseases that often require glucocorticoids				
Single delivery	11 (20.8)	2,653 (23.2)	0.9 [0.4, 1.7]	0.747
Twin delivery	1 (16.7)	23 (21.3)	0.7 [0.0, 7.1]	1.000
Skin diseases that often require glucocorticoids				
Single delivery	19 (35.8)	4,867 (42.5)	0.8 [0.4, 1.4]	0.404
Twin delivery	0 (0.0)	48 (44.4)	0.0 [0.0, 0.9]	0.039
Rheumatoid arthritis				
Single delivery	2 (3.8)	74 (0.6)	6.0 [0.7, 23.7]	0.048
Twin delivery	1 (16.7)	0 (0.0)	NE	0.053
Systemic lupus erythematosus				
Single delivery	0 (0.0)	46 (0.4)	0.0 [0.0, 14.9]	1.000
Twin delivery	1 (16.7)	0 (0.0)	NE	0.053
Dermatopolymyositis				
Single delivery	0 (0.0)	3 (0.0)	0.0 [0.0, 374.8]	1.000
Twin delivery	0 (0.0)	0 (0.0)	NE	
Ovulation disorder				
Single delivery	19 (35.8)	2,512 (21.9)	2.0 [1.1, 3.6]	0.020
Twin delivery	3 (50.0)	66 (61.1)	0.6 [0.1, 5.0]	0.679
Female infertility due to other than ovulation disorder				
Single delivery	20 (37.7)	3,143 (27.5)	1.6 [0.9, 2.9]	0.122
Twin delivery	3 (50.0)	66 (61.1)	0.6 [0.1, 5.0]	0.679

CI, confidence interval; NE, not possible to estimate; PLO, pregnancy and lactation-associated osteoporosis

^a^*P* value for Fisher’s exact test

### Comparison of diseases in children of PLO and control mothers

A comparison of the characteristics of the children born to PLO mothers and control mothers is shown in Table S6. Observation periods were significantly longer in the controls than in the case children, or were comparable between the two groups.

A comparison of neonatal diseases between the two groups is shown in Table 5. Both groups had comparable frequencies of all investigated diseases. There were trends toward a higher frequency of birth asphyxia in singletons (*P* = 0.072) and low birth weight in twins (*P* = 0.070) in the case children compared with the controls; however, these differences did not reach statistical significance.

**Table 5:** Neonatal diseases in children born to mothers in the PLO and control groups

	Children of PLO mothers, *n* (%)	Control, *n* (%)	Risk ratio^a^ [95% CI]	*P* value^b^
Preterm infants				
Single delivery	2 (3.8)	320 (2.8)	1.4 [0.4, 4.6]	0.661
Twin delivery	5 (41.7)	64 (29.6)	1.4 [0.6, 2.4]	0.519
Low birth weight				
Single delivery	1 (1.9)	663 (5.8)	0.3 [0.1, 1.7]	0.371
Twin delivery	10 (83.3)	116 (53.7)	1.6 [1.0, 1.9]	0.070
Birth asphyxia				
Single delivery	4 (7.5)	337 (2.9)	2.6 [1.0, 6.1]	0.072
Twin delivery	0 (0.0)	33 (15.3)	0.0 [0.0, 1.6]	0.223
Neonatal hypothermia				
Single delivery	1 (1.9)	119 (1.0)	1.8 [0.3, 9.6]	0.427
Twin delivery	0 (0.0)	6 (2.8)	0.0 [0.0, 9.7]	1.000
Neonatal hypoglycemia				
Single delivery	3 (5.7)	629 (5.5)	1.0 [0.4, 2.8]	0.767
Twin delivery	4 (33.3)	51 (23.6)	1.4 [0.6, 2.8]	0.490
Neonatal respiratory disorders				
Single delivery	5 (9.4)	953 (8.3)	1.1 [0.5, 2.4]	0.801
Twin delivery	2 (16.7)	64 (29.6)	0.6 [0.2, 1.6]	0.516
Neonatal jaundice				
Single delivery	5 (9.4)	1,283 (11.2)	0.8 [0.4, 1.8]	0.829
Twin delivery	2 (16.7)	21 (9.7)	1.7 [0.5, 5.1]	0.346

PLO, pregnancy and lactation-associated osteoporosis

^a^The risk ratio compares the risk of developing a particular neonatal disease between the PLO and control groups. The reference is the risk for the control group (risk ratio = 1)

^b^*P* value for Fisher’s exact test

A comparison of the diseases occurring throughout childhood is shown in Table 6. The frequencies of obesity and malnutrition in singletons were significantly greater in the case children than in the controls; however, each of these conditions was observed in only one child. No significant differences in the frequencies of other diseases were observed between the two groups.

**Table 6:** Diseases during childhood in children born to mothers in the PLO and control groups

	Children of PLO mothers, *n* (%)	Control, *n* (%)	Hazard ratio^a^ [95% CI]	*P* value^b^
Vitamin D deficiency				
Single delivery	0 (0.0)	91 (0.8)	NE	0.522
Twin delivery	0 (0.0)	11 (5.1)	NE	0.426
Obesity				
Single delivery	1 (1.9)	57 (0.5)	6.7 [0.9, 48.2]	0.030
Twin delivery	0 (0.0)	1 (0.5)	NE	0.746
Malnutrition				
Single delivery	1 (1.9)	37 (0.3)	6.8 [0.9, 49.6]	0.028
Twin delivery	0 (0.0)	3 (1.4)	NE	0.682
Disorders of psychological development				
Single delivery	5 (9.4)	1,033 (9.0)	1.3 [0.6, 3.2]	0.513
Twin delivery	0 (0.0)	37 (17.1)	NE	0.172
Lack of expected physiological development				
Single delivery	5 (9.4)	1,005 (8.8)	1.1 [0.5, 2.7]	0.814
Twin delivery	2 (16.7)	20 (9.3)	2.2 [0.5, 9.5]	0.273
Fracture				
Single delivery	3 (5.7)	1,046 (9.1)	1.0 [0.3, 3.1]	0.989
Twin delivery	2 (16.7)	26 (12.0)	1.0 [0.2, 4.5]	0.956
Hospitalization due to infectious diseases				
Single delivery	12 (22.6)	2,154 (18.8)	1.4 [0.8, 2.4]	0.277
Twin delivery	2 (16.7)	46 (21.3)	0.8 [0.2, 3.3]	0.741

CI, confidence interval; NE, not possible to estimate; PLO, pregnancy and lactation-associated osteoporosis

^a^The hazard ratio compares the hazard of developing a particular disease between the PLO and control groups. The reference is the hazard for the control group (hazard ratio = 1)

^b^*P* value for log-rank test

## Discussion

To the best of our knowledge, this is the first report to investigate the effects of maternal PLO on child health. Although we hypothesized that children born to mothers with PLO would develop certain conditions due to an insufficient calcium supply, our results provided little evidence of adverse effects on neonatal health. While there were trends toward a higher frequency of birth asphyxia and low birth weight in the case children compared with the controls, these differences did not reach statistical significance. Furthermore, the comparison of diseases throughout childhood showed little difference between the two groups. These results may indicate that physiological changes during pregnancy and lactation [13–16] are adequate for child growth, even under the impaired bone homeostasis associated with PLO, although reliable data on the amount of calcium supplied to these children remain unavailable.

Alternatively, considering that the mortality and morbidity of children depend largely on prematurity and birth weight [25], the comparable prevalence of preterm infants and low birth weight between the two groups might explain the similar prevalence of other pediatric conditions. Furthermore, since low maternal BMI and poor gestational weight gain are established predictors of neonatal prematurity and low birth weight [19–21], the comparable prevalence of these outcomes between the two groups might be associated with the relatively low BMI of the control mothers (median 20 kg/m^2^), notwithstanding that BMI values were significantly lower in PLO mothers in the single delivery group. In contrast, a previous study [19] included only controls with a normal BMI (> 19.8, < 26 kg/m^2^) to demonstrate the association of low maternal BMI (≤ 19.8 kg/m^2^) with neonatal prematurity and low birth weight. Additionally, it has been reported that gestational weight gain has a greater effect on birth weight than maternal BMI [21], although gestational weight gain could not be assessed in the present study.

Furthermore, our study presented several epidemiological findings regarding PLO. The most common PLO fractures were vertebral fractures. When diseases potentially causing musculoskeletal complaints were investigated, nearly half of the PLO patients were found to have such conditions and had received treatment for them before their fracture diagnosis. These findings suggest that a delayed diagnosis occurred in at least some of these cases [9, 26, 27].

The incidence of PLO in the present study, at 374 per million deliveries, is lower than our prior estimate of 460 per million deliveries [2]. This difference is likely due to the use of a new dataset and stricter inclusion criteria for PLO mothers, specifically, women without imaging or treatment in the same month as their fractures were excluded to ensure that only confirmed fracture cases were analyzed. However, this incidence remains much greater than the values estimated by other authors [8, 9] even when the period for PLO is restricted to fractures occurring from -3 M to + 2 M after delivery.

The discrepancy in the incidence of PLO between a previous case series by Orhadje et al. [9] and the present study might stem from the difference between medical record-based and database-driven studies. We agree with their opinion that the incidence of PLO at 4–8 per million pregnancies in a past study [8] lacked robust data. Nevertheless, their estimate of 6.8 per 100,000 pregnancies is lower than that in the present study, despite the temporal range of fractures being larger in their study than in the present one. Although they clearly explained their estimation process, the possibility of unreported cases cannot be ruled out.

Additionally, a previous estimate of PLO incidence using the Japanese Diagnostic Procedure Combination (DPC) database may be unreliable [28]. While young patients who develop low-trauma vertebral fractures—the most common PLO fracture—are generally treated on an outpatient basis, DPC database covers only inpatients. Furthermore, in their study, vertebral fractures accounted for only 40% of cases, which is lower than in the previous studies on PLO [1–3, 22, 23], and most patients had multiple fractures, suggesting the inclusion of high-energy trauma cases.

Meanwhile, to the best of our knowledge, the present study is the first to demonstrate that multiple pregnancy is a strong predictor of PLO. We found a nine-fold increased risk of PLO compared with single deliveries, likely due to the use of a large-scale dataset linking mother and child data. When restricted to the six months from -3M to + 2M after delivery, the incidence of PLO in multiple deliveries was 22 times greater than in single deliveries. This indicates that fetal demand for calcium and mechanical loading on the maternal spine in multiple pregnancies increase drastically toward delivery. In our previous study, we considered that all PLO patients had given birth to singletons because none were assigned the ICD-10 code of multiple delivery (O84) [2]. However, some of those patients had been assigned the multiple pregnancy code (O30). The use of a dataset consisting only of maternal records may have misled readers regarding this matter.

As mentioned earlier, BMI values were significantly lower in PLO mothers with single deliveries; however, the prevalence of underweight (BMI < 18.5 kg/m^2^, according to the WHO classification) was not significantly higher than in controls. These results are consistent with the findings of a previous study [8] in terms of the overall trend toward lower BMI in the PLO group. The relatively low BMI of the control group (median: approximately 20 kg/m^2^) in the present study might reflect findings from a recent article reporting that many young Japanese women have a strong desire to be thin [29].

Furthermore, the present study identified the maternal age group at high risk of PLO as 40 years or older, providing a more specific threshold than previous reports that simply claimed advanced maternal age as a risk factor [2, 9–11, 28]. This finding is compatible with general knowledge regarding the epidemiology of premenopausal osteoporosis; namely, that a natural decrease in bone mineral density (BMD) and a natural increase in fracture incidence begin as early as the midpoint of reproductive age [30].

The potential risks of UFH treatment and ovulation disorders for PLO were also predictable. In Japan, unlike UFH, self-injection of low-molecular-weight heparin (LMWH) [9] is not covered by health insurance. Thus, for women with a history of thromboembolic diseases or those with anti-phospholipid antibodies and preceding recurrent pregnancy loss, self-injection of UFH is recommended until just before delivery, despite reports that UFH has a more detrimental effect on bone than LMWH [31, 32]. In addition, the present results suggest that RA might be a predisposing factor for PLO, even though GCs (which play an important role in bone loss of RA patients) and other diseases requiring GCs failed to show a significant risk for PLO. In the case of RA, the systemic effects of the disease itself might account for these results [33]. Furthermore, it is likely that few patients with serious comorbidities were included in the present study cohort, which consisted parous women of reproductive age.

The present results regarding the predisposing factors for PLO provide clear warnings regarding current reproductive technologies that have brought substantial benefits to many women. Progress in assisted reproductive technology, including improved ovulation induction, has encouraged slender women with ovulation disorders, as well as women reaching the latter half of their reproductive age. Furthermore, heparin has given hope to young patients with a high risk of thromboembolism. Nevertheless, physicians should provide women who have predisposing factors for PLO—including a family history of osteoporosis—with appropriate information before starting ovulation induction, which often leads to multiple deliveries.

One strength of the present study is the use of a large-scale claims dataset linking mother and child data, notwithstanding the limited evidence of adverse effects of maternal PLO on the health of their children. Because PLO is a rare and often underdiagnosed condition, findings from medical record-based studies [5, 9, 26] and online questionnaire-based studies [27, 34] typically depend on physician awareness and the active response of participants, respectively. Our dataset allowed us to investigate the epidemiology of PLO—which is strongly related to reproduction and parenting—independently of these subjective factors.

Nevertheless, the present study has several limitations. First, the database does not provide clinical details such as the onset of pain, gestational weight gain, lactation period, BMD values, or family history of osteoporosis. Although we developed a method to estimate the actual fracture time in a database study, it remains difficult to compare our results directly with those of clinical studies. Second, to ensure a sufficient sample size for BMI analysis, we considered BMI values both before and after pregnancy. This approach was necessary because more than half of the women in both groups lacked BMI records, and most mothers who had records for both periods provided similar values (data not shown). Third, temporal trends and geographical differences may influence the incidence of this condition.

In conclusion, our findings provided little evidence that maternal PLO has deleterious effects on the health of the children. These results support the robustness of maternal physiological adaptations in calcium and bone metabolism, which ensure sufficient calcium for fetal and infant growth even under the impaired bone homeostasis associated with PLO. Furthermore, this study estimated the incidence of PLO at 374 per million deliveries, a figure that likely brings us closer to the actual incidence. Our results also suggest that a substantial number of PLO diagnoses may have been delayed. Multiple deliveries, maternal age of 40 years or older, low BMI, use of UFH, and ovulation disorder were identified as predictors for PLO. Therefore, physicians should provide women who wish to conceive with appropriate information regarding PLO risk before undergoing ovulation induction, which frequently leads to multiple deliveries.

## Supplementary Information

Below is the link to the electronic supplementary material.

Supplementary file1 (DOCX 70 KB)

Supplementary file2 (DOCX 56 KB)

Supplementary file3 (DOCX 124 KB)

Supplementary file4 (DOCX 57 KB)

Supplementary file5 (DOCX 56 KB)

Supplementary file6 (DOCX 59 KB)

Supplementary file7 (DOCX 58 KB)

## Data Availability

The data that support the findings of this study are available from the corresponding author (KK and ST) on reasonable request.
