# Supplementary material for: Effects of pregnancy and lactation-associated osteoporosis on children: analysis using linked mother and child data
Source: J Bone Miner Metab. 2026 Apr 11;44(4):501–12. doi: 10.1007/s00774-026-01720-4 (PMC13468983; doi:10.1007/s00774-026-01720-4)
Supplement: Supplementary file 1 [file 774_2026_1720_MOESM1_ESM.docx]

**Effects of pregnancy and lactation-associated osteoporosis on children: Analysis using linked mother and child data**

*Journal of Bone and Mineral Metabolism*

Kyoko Kasahara, Shunichiro Tsuji*, Taku Kawasaki, and Takashi Murakami

***Corresponding author**

Shunichiro Tsuji

The Department of Obstetrics and Gynecology, Shiga University of Medical Science, Seta Tsukinowa-cho, Otsu, Shiga, Japan

e-mail: tsuji002@belle.shiga-med.ac.jp

**Supplemental Table 1** List of codes investigated

| **Variable** | **Type of code** | **Code starts with** | **Period** | **Note** |
| --- | --- | --- | --- | --- |
| **Fragility fracture of mothers** |  |  |  |  |
| Vertebral fracture |  |  |  |  |
| Thoracic fracture | ICD-10 code | S220, S221 | −5M to +12M^c^ |  |
| Lumbar fracture | ICD-10 code | S320, S327 | −5M to +12M^c^ |  |
| Fracture of spine, miscellaneous | ICD-10 code | T021, T08- | −5M to +12M^c^ |  |
| Pubic fracture | ICD-10 code | S325 | −5M to +12M^c^ |  |
| Pelvic fracture excluding pubic fracture | ICD-10 code | S328 | −5M to +12M^c^ |  |
| Proximal humeral fracture | ICD-10 code | S422 | −5M to +12M^c^ |  |
| Distal radius fracture | ICD-10 code | S525 | −5M to +12M^c^ |  |
| Proximal femoral fracture | ICD-10 code | S720, S721, S722 | −5M to +12M^c^ |  |
| Sequelae of fracture |  |  |  |  |
| Old vertebral fracture | ICD-10 code | T911 | −5M to +12M^c^ |  |
| Old proximal femoral fracture | ICD-10 code | T931 | −5M to +12M^c^ |  |
| **Diseases related to maternal fracture risk** |  |  |  |  |
| Secondary malignant neoplasm of bone | ICD-10 code | C795 | <−5M^d^ |  |
| Idiopathic thrombocytopenic purpura | ICD-10 code | D693 | <−5M^d^ |  |
| Thyroid gland disorders | ICD-10 code | E00-E07 | <−5M^d^ |  |
| Diabetes mellitus | ICD-10 code | E10-E14 | <−5M^d^ |  |
| Eating disorders | ICD-10 code | F50 | <−5M^d^ |  |
| Epilepsy | ICD-10 code | G40, G41 | <−5M^d^ |  |
| Respiratory diseases that often require glucocorticoids |  |  |  |  |
| Acute laryngitis (croup) and epiglottitis | ICD-10 code | J05 | <−5M^d^ |  |
| Chronic sinusitis | ICD-10 code | J32 | <−5M^d^ |  |
| Asthma | ICD-10 code | J45, J46 | <−5M^d^ |  |
| Skin diseases that often require glucocorticoids |  |  |  |  |
| Dermatitis and eczema | ICD-10 code | L20-L30 | <−5M^d^ |  |
| Urticaria and eczema | ICD-10 code | L50-L54 | <−5M^d^ |  |
| Rheumatoid arthritis | ICD-10 code | M053, M059, M060, M068, M069 | <−5M^d^ |  |
| Systemic lupus erythematosus | ICD-10 code | M32 | <−5M^d^ |  |
| Dermatopolymyositis | ICD-10 code | M33 | <−5M^d^ |  |
| Absent, scanty, and rare menstruation^a^ | ICD-10 code | N91 | <−5M^d^ |  |
| Female infertility associated with anovulation^a^ | ICD-10 code | N970 | <−5M^d^ |  |
| Female infertility of tubal origin^b^ | ICD-10 code | N971 | <−5M^d^ |  |
| Female infertility of uterine origin^b^ | ICD-10 code | N972 | <−5M^d^ |  |
| Female infertility of cervical origin^b^ | ICD-10 code | N973 | <−5M^d^ |  |
| Female infertility of other origin^b^ | ICD-10 code | N978 | <−5M^d^ |  |
| Female infertility unspecified^b^ | ICD-10 code | N979 | <−5M^d^ |  |
| Osteogenesis imperfecta | ICD-10 code | Q780 | <−5M^d^ |  |
| **Diseases of mothers that could cause musculoskeletal complaints requiring treatment** |  |  |  |  |
| Sprain and strain of thoracic spine | ICD-10 code | S233 | −8M to +11M^e^ |  |
| Sprain and strain of lumbar spine | ICD-10 code | S335 | −8M to +11M^e^ |  |
| Spondylosis without myelopathy or radiculopathy | ICD-10 code | M478 | −8M to +11M^e^ |  |
| Intervertebral disc disorders, unspecified | ICD-10 code | M519 | −8M to +11M^e^ |  |
| Low back pain | ICD-10 code | M545 | −8M to +11M^e^ |  |
| Pain localized to lower abdomen | ICD-10 code | R103 | −8M to +11M^e^ |  |
| **Neonatal infant diseases** |  |  |  |  |
| Neonatal hypoglycemia | ICD-10 code | E161, E162, P704 | 0M or 1M^f^ | Inpatient |
| Low birth weight | ICD-10 code | P070, P071 | 0M^g^ | Inpatient |
| Preterm infants | ICD-10 code | P072, P073 | 0M^g^ | Inpatients |
| Birth asphyxia | ICD-10 code | P21 | 0M or 1M^f^ | Inpatient |
| Neonatal respiratory disorders | ICD-10 code | P22-P28 | 0M or 1M^f^ | Inpatient |
| Neonatal jaundice | ICD-10 code | P57-59 | 0M or 1M^f^ | Inpatient |
| Hyperbilirubinemia | Standard disease code | 7746001 | 0M or 1M^f^ | Inpatient |
| Neonatal hypothermia | ICD-10 code | P808, T68 | 0M or 1M^f^ | Inpatient |
| **Diseases during childhood** |  |  |  |  |
| Malnutrition | ICD-10 code | E40-E46 | Anytime |  |
| Vitamin D deficiency, rickets | ICD-10 code | E55 | Anytime |  |
| Obesity | ICD-10 code | E66 | Anytime |  |
| Disorders of psychological development | ICD-10 code | F80-F89 | Anytime |  |
| Lack of expected normal physiological development | ICD-10 code | R62 | Anytime |  |
| Fractures | ICD-10 code | S02, S22, S32, S42, S52, S62, S72, S82, S92 | Anytime |  |
| Hospitalization due to infectious diseases |  |  |  |  |
| Certain infectious and parasitic diseases | ICD-10 code | A00-B99 | Anytime | Inpatient |
| Inflammatory diseases of the central nervous system | ICD-10 code | G00-G09 | Anytime | Inpatient |
| Acute upper respiratory infections | ICD-10 code | J00-J06 | Anytime | Inpatient |
| Influenza and pneumonia | ICD-10 code | J09-J18 | Anytime | Inpatient |
| Other acute lower respiratory infections | ICD-10 code | J20-J22 | Anytime | Inpatient |
| Suppurative and necrotic conditions of lower respiratory tract | ICD-10 code | J85-J86 | Anytime | Inpatient |
| Infections of the skin and subcutaneous tissue | ICD-10 code | L00-L08 | Anytime | Inpatient |
| Urinary tract infection | ICD-10 code | N390 | Anytime | Inpatient |
| **Imaging examinations** |  |  |  |  |
| Radiography | Japanese standardized procedure code | E002 | −5M to +12M^c^ |  |
| Computed tomography | Japanese standardized procedure code | E200 | −5M to +12M^c^ |  |
| Magnetic resonance imaging | Japanese standardized procedure code | E202 | −5M to +12M^c^ |  |
| **Back braces** |  |  |  |  |
| Lumbar fixation belt | Japanese standardized procedure code | J119-2 | −8M to +12M^h^ |  |
| Orthotic creation | Japanese standardized procedure code | J129-4 | −8M to +12M^h^ |  |
| **Drugs of mothers** |  |  |  |  |
| Anticoagulants |  |  |  |  |
| Unfractionated heparin | EphMRA code | B01B1 | ≤0M^i^ | ≥56A, 2M^j^ |
| Warfarin | WHO code | B01AA03 | <−5M^d^ | ≥28D, 2M^k^ |
| Glucocorticoids |  |  |  |  |
| Injection | EphMRA code | H02A1 | <−5M^d^ | ≥3D, 2M^l^ |
| Oral administration | EphMRA code | H02A2 | <−5M^d^ | ≥28D, 2M^k^ |
| Analgesics, oral | |  |  |  |
| Indomethacin | WHO code | M01AB01 | −8M to +12M^h^ |  |
| Diclofenac | WHO code | M01AB05 | −8M to +12M^h^ |  |
| Etodolac | WHO code | M01AB08 | −8M to +12M^h^ |  |
| Lornoxicam | WHO code | M01AC05 | −8M to +12M^h^ |  |
| Meloxicam | WHO code | M01AC06 | −8M to +12M^h^ |  |
| Propionic acid, e.g. Loxoprofen | WHO code | M01AE | −8M to +12M^h^ |  |
| Ibuprofen | WHO code | M01AE01 | −8M to +12M^h^ |  |
| Ketoprofen | WHO code | M01AE03 | −8M to +12M^h^ |  |
| Mefenamic acid | WHO code | M01AG01 | −8M to +12M^h^ |  |
| Celecoxib | WHO code | M01AH01 | −8M to +12M^h^ |  |
| Acetaminophen | WHO code | N02BE01 | −8M to +12M^h^ |  |
| Analgesics, cold patch | |  |  |  |
| Felbinac | WHO code | M02AA08 | −8M to +12M^h^ |  |
| Ketoprofen | WHO code | M02AA10 | −8M to +12M^h^ |  |
| Diclofenac | WHO code | M02AA15 | −8M to +12M^h^ |  |
| Flurbiprofen | WHO code | M02AA19 | −8M to +12M^h^ |  |
| Indomethacin | WHO code | M02AA23 | −8M to +12M^h^ |  |
| Propionic acid, e.g. loxoprofen | WHO code | M02AA31 | −8M to +12M^h^ |  |
| Antiseizure medications related to osteoporosis |  |  |  |  |
| Phenobarbital | WHO code | N03AA02 | <−5M^d^ | ≥28D, 2M^k^ |
| Primidone | WHO code | N03AA03 | <−5M^d^ | ≥28D, 2M^k^ |
| Phenytoin | WHO code | N03AB02 | <−5M^d^ | ≥28D, 2M^k^ |
| Carbamazepine | WHO code | N03AF01 | <−5M^d^ | ≥28D, 2M^k^ |
| Valproic acid | WHO code | N03AG01 | <−5M^d^ | ≥28D, 2M^k^ |

ICD-10, International Classification of Diseases, 10th revision; EphMRA, European Pharmaceutical Market Research Association; WHO, World Health Organization

^a^Combined as “ovulation disorder”

^b^Combined as “female infertility due to other than ovulation disorder”

^c^Between 5 months before and 12 months after delivery

^d^Prior to 5 months before delivery

^e^Between 8 months before and 11 months after delivery

^f^The same or next month of birth

^g^The same month of birth

^h^Between 8 months before and 12 months after delivery

^i^Before or the same month of delivery

^j^More than 56 ampoules during 2 consecutive months

^k^More than 28 prescription days during 2 consecutive months

^l^More than 3 treatment days during 2 consecutive months.
