# Supplementary material for: Effects of pregnancy and lactation-associated osteoporosis on children: analysis using linked mother and child data
Source: J Bone Miner Metab. 2026 Apr 11;44(4):501–12. doi: 10.1007/s00774-026-01720-4 (PMC13468983; doi:10.1007/s00774-026-01720-4)
Supplement: Supplementary file 2 [file 774_2026_1720_MOESM2_ESM.docx]

**Effects of pregnancy and lactation-associated osteoporosis on children: Analysis using linked mother and child data**

*Journal of Bone and Mineral Metabolism*

Kyoko Kasahara, Shunichiro Tsuji*, Taku Kawasaki, and Takashi Murakami

***Corresponding author**

Shunichiro Tsuji

The Department of Obstetrics and Gynecology, Shiga University of Medical Science, Seta Tsukinowa-cho, Otsu, Shiga, Japan

e-mail: tsuji002@belle.shiga-med.ac.jp

**Supplemental Table 2** Characteristics of mothers with PLO

| **Characteristic** | **Total**  ***n* = 59** | **Single delivery**  ***n* = 53** | **Twin delivery**  ***n* = 6** |
| --- | --- | --- | --- |
| Maternal age, y, median (range) | 34 (23, 44) | 35 (23, 44) | 31 (27, 38) |
| Maternal age, categorized |  |  |  |
| <30 y, *n* (%) | 14 (23.7) | 12 (22.6) | 2 (33.3) |
| 30-39 y, *n* (%) | 36 (61.0) | 32 (60.4) | 4 (66.7) |
| 40-47 y, *n* (%) | 9 (15.3) | 9 (17.0) | 0 (0.0) |
| Fragility fracture |  |  |  |
| Vertebral fracture, *n* (%) | 49 (83.1) | 45 (84.9) | 4 (66.7) |
| Pubic fracture, *n* (%) | 8 (13.6) | 6 (11.3) | 2 (33.3) |
| Pelvic fracture  excluding the pubis, *n* (%) | 1 (1.7) | 1 (1.9) | 0 |
| Proximal femoral fracture, *n* (%) | 1 (1.7) | 1 (1.9) | 0 |
