# Supplementary material for: Effects of pregnancy and lactation-associated osteoporosis on children: analysis using linked mother and child data
Source: J Bone Miner Metab. 2026 Apr 11;44(4):501–12. doi: 10.1007/s00774-026-01720-4 (PMC13468983; doi:10.1007/s00774-026-01720-4)
Supplement: Supplementary file 3 [file 774_2026_1720_MOESM3_ESM.docx]

**Effects of pregnancy and lactation-associated osteoporosis on children: Analysis using linked mother and child data**

*Journal of Bone and Mineral Metabolism*

Kyoko Kasahara, Shunichiro Tsuji*, Taku Kawasaki, and Takashi Murakami

***Corresponding author**

Shunichiro Tsuji

The Department of Obstetrics and Gynecology, Shiga University of Medical Science, Seta Tsukinowa-cho, Otsu, Shiga, Japan

e-mail: [tsuji002@belle.shiga-med.ac.jp](mailto:tsuji002@belle.shiga-med.ac.jp)

**Supplementary Fig. 1** Temporal distribution of PLO fracture diagnosis

Fracture diagnoses are presented monthly by temporal relationship with delivery.

(a) All 59 PLO factures

(b) Thirty-two PLO fractures, excluding the 27 cases in which treatment for musculoskeletal complaints was administered prior to fracture diagnosis.

PLO: pregnancy and lactation-associated osteoporosis
