# Supplementary material for: Effects of pregnancy and lactation-associated osteoporosis on children: analysis using linked mother and child data
Source: J Bone Miner Metab. 2026 Apr 11;44(4):501–12. doi: 10.1007/s00774-026-01720-4 (PMC13468983; doi:10.1007/s00774-026-01720-4)
Supplement: Supplementary file 4 [file 774_2026_1720_MOESM4_ESM.docx]

**Effects of pregnancy and lactation-associated osteoporosis on children: Analysis using linked mother and child data**

*Journal of Bone and Mineral Metabolism*

Kyoko Kasahara, Shunichiro Tsuji*, Taku Kawasaki, and Takashi Murakami

***Corresponding author**

Shunichiro Tsuji

The Department of Obstetrics and Gynecology, Shiga University of Medical Science, Seta Tsukinowa-cho, Otsu, Shiga, Japan

e-mail: tsuji002@belle.shiga-med.ac.jp

**Supplemental Table 3** Temporal relationship between musculoskeletal complaints and fracture diagnosis in the 27 PLO mothers who had musculoskeletal complaints requiring treatment

|  | | **Total,**  ***n*** | **Temporal relationship between musculoskeletal**  **complaints and fracture diagnosis (months)** | | | | | | | |
| --- | --- | --- | --- | --- | --- | --- | --- | --- | --- | --- |
|  |  |  | **-1** | **-2** | **-3** | **-4** | **-5** | **-6** | **-7** | **-8** |
| **Temporal relationship between fracture diagnosis and delivery (months)** | **0** | 1 | 1 | 0 | 0 | 0 | 0 | 0 | 0 | 0 |
|  | **+1** | 2 | 1 | 0 | 1 | 0 | 0 | 0 | 0 | 0 |
|  | **+2** | 8 | 4 | 4 | 0 | 0 | 0 | 0 | 0 | 0 |
|  | **+3** | 7 | 4 | 0 | 2 | 0 | 0 | 1 | 0 | 0 |
|  | **+4** | 4 | 1 | 0 | 0 | 3 | 0 | 0 | 0 | 0 |
|  | **+5** | 0 | 0 | 0 | 0 | 0 | 0 | 0 | 0 | 0 |
|  | **+6** | 2 | 0 | 0 | 0 | 0 | 0 | 2 | 0 | 0 |
|  | **+7** | 2 | 0 | 0 | 0 | 0 | 0 | 1 | 1 | 0 |
|  | **+8** | 0 | 0 | 0 | 0 | 0 | 0 | 0 | 0 | 0 |
|  | **+9** | 1 | 0 | 0 | 0 | 0 | 0 | 0 | 0 | 1 |

The numbers of musculoskeletal complaints in the month of delivery are marked as gray.

PLO, pregnancy and lactation-associated osteoporosis.
