# Supplementary material for: Effects of pregnancy and lactation-associated osteoporosis on children: analysis using linked mother and child data
Source: J Bone Miner Metab. 2026 Apr 11;44(4):501–12. doi: 10.1007/s00774-026-01720-4 (PMC13468983; doi:10.1007/s00774-026-01720-4)
Supplement: Supplementary file 5 [file 774_2026_1720_MOESM5_ESM.docx]

**Effects of pregnancy and lactation-associated osteoporosis on children: Analysis using linked mother and child data**

*Journal of Bone and Mineral Metabolism*

Kyoko Kasahara, Shunichiro Tsuji*, Taku Kawasaki, and Takashi Murakami

***Corresponding author**

Shunichiro Tsuji

The Department of Obstetrics and Gynecology, Shiga University of Medical Science, Seta Tsukinowa-cho, Otsu, Shiga, Japan

e-mail: tsuji002@belle.shiga-med.ac.jp

**Supplemental Table 4** Incidence of PLO as fragility fractures between 3 months before and 2 months after delivery

| **Cohort** | **Deliveries, *n*** | **PLO, *n* (%)** | **PLO incidence^a^ [95% CI]** | **Risk ratio [95% CI]** | ***P* value^b^** |
| --- | --- | --- | --- | --- | --- |
| Full cohort | 157,823 | 28 (0.018) | 177 [122, 257] |  |  |
| Single delivery | 155,927 | 22 (0.014) | 141 [93, 214] | Reference |  |
| Multiple delivery | 1,896 | 6 (0.316) | 3,165 [1,422, 7,044] | 22.4 [9.1, 55.3] | <0.001 |

CI, confidence interval; PLO, pregnancy and lactation-associated osteoporosis

^a^PLO incidence per 1,000,000 deliveries

^b^*P* value for Poisson regression
