# Supplementary material for: Effects of pregnancy and lactation-associated osteoporosis on children: analysis using linked mother and child data
Source: J Bone Miner Metab. 2026 Apr 11;44(4):501–12. doi: 10.1007/s00774-026-01720-4 (PMC13468983; doi:10.1007/s00774-026-01720-4)
Supplement: Supplementary file 6 [file 774_2026_1720_MOESM6_ESM.docx]

**Effects of pregnancy and lactation-associated osteoporosis on children: Analysis using linked mother and child data**

*Journal of Bone and Mineral Metabolism*

Kyoko Kasahara, Shunichiro Tsuji*, Taku Kawasaki, and Takashi Murakami

***Corresponding author**

Shunichiro Tsuji

The Department of Obstetrics and Gynecology, Shiga University of Medical Science, Seta Tsukinowa-cho, Otsu, Shiga, Japan

e-mail: tsuji002@belle.shiga-med.ac.jp

**Supplemental Table 5** Characteristics of mothers in the PLO and control groups

|  | **Mothers with PLO** | **Control** | ***P* value**^a^ |
| --- | --- | --- | --- |
| **Single delivery** |  |  |  |
| Mothers, *n* | 53 | 11,448 |  |
| Maternal age, y, median (range) | 35 (23, 44) | 35 (23, 44) |  |
| Maternal age, categorized |  |  |  |
| < 30 y, *n* (%) | 12 (22.6) | 2,592 (22.6) |  |
| 30-39 y, *n* (%) | 32 (60.4) | 6,912 (60.4) |  |
| 40-47 y, *n* (%) | 9 (17.0) | 1,944 (17.0) |  |
| Observation period before delivery, months, median (range) | 33 (13, 127) | 30 (13, 172) | 0.922 |
| Observation period before delivery, categorized |  |  | 0.959 |
| < 1 y, *n* (%) | 0 (0) | 0 (0) |  |
| 1–2 y, *n* (%) | 32 (60) | 6,986 (61) |  |
| 3–4 y, *n* (%) | 14 (26) | 3,059 (27) |  |
| ≥5 y, *n* (%) | 7 (13) | 1,403 (12) |  |
| Observation period after delivery, months, median (range) | 47 (15, 139) | 70 (15, 192) | <0.001 |
| Observation period after delivery, categorized |  |  | 0.001 |
| < 1 y, *n* (%) | 0 (0) | 0 (0) |  |
| 1–2 y, *n* (%) | 19 (36) | 1,801 (16) |  |
| 3–4 y, *n* (%) | 11 (21) | 2,737 (24) |  |
| ≥5 y, *n* (%) | 23 (43) | 6,910 (60) |  |
| **Twin delivery** |  |  |  |
| Mothers, *n* | 6 | 108 |  |
| Maternal age, years, median (range) | 31 (27, 38) | 31 (27, 38) |  |
| Maternal age, categorized |  |  |  |
| < 30 y, *n* (%) | 2 (33.3) | 36 (33.3) |  |
| 30-39 y, *n* (%) | 4 (66.7) | 72 (66.7) |  |
| 40-49 y, *n* (%) | 0 (0.0) | 0 (0.0) |  |
| Observation period before delivery, months, median (range) | 27 (14, 55) | 29 (13, 99) | 0.814 |
| Observation period before delivery, categorized |  |  | 1.000 |
| < 1 y, *n* (%) | 0 (0) | 0 (0) |  |
| 1–2 y, *n* (%) | 5 (83) | 75 (69) |  |
| 3–4 y, *n* (%) | 1 (17) | 27 (25) |  |
| ≥5 y, *n* (%) | 0 (0) | 6 (6) |  |
| Observation period after delivery, months, median (range) | 62 (15, 156) | 76 (16, 178) | 0.727 |
| Observation period after delivery, categorized |  |  | 0.277 |
| < 1 y, *n* (%) | 0 (0) | 0 (0) |  |
| 1–2 y, *n* (%) | 2 (33) | 13 (12) |  |
| 3–4 y, *n* (%) | 1 (17) | 23 (21) |  |
| ≥5 y, *n* (%) | 3 (50) | 72 (67) |  |

PLO, pregnancy and lactation-associated osteoporosis

^a^Mann-Whitney *U* test and Fisher’s exact test for continuous and categorical variables, respectively
