# Supplementary material for: Effects of pregnancy and lactation-associated osteoporosis on children: analysis using linked mother and child data
Source: J Bone Miner Metab. 2026 Apr 11;44(4):501–12. doi: 10.1007/s00774-026-01720-4 (PMC13468983; doi:10.1007/s00774-026-01720-4)
Supplement: Supplementary file 7 [file 774_2026_1720_MOESM7_ESM.docx]

**Effects of pregnancy and lactation-associated osteoporosis on children: Analysis using linked mother and child data**

*Journal of Bone and Mineral Metabolism*

Kyoko Kasahara, Shunichiro Tsuji*, Taku Kawasaki, and Takashi Murakami

***Corresponding author**

Shunichiro Tsuji

The Department of Obstetrics and Gynecology, Shiga University of Medical Science, Seta Tsukinowa-cho, Otsu, Shiga, Japan

e-mail: tsuji002@belle.shiga-med.ac.jp

**Supplemental Table 6** Characteristics of children born to mothers in the PLO and control groups

|  | **Children of PLO mothers** | **Control** | ***P* value**^a^ |
| --- | --- | --- | --- |
| **Single delivery** |  |  |  |
| Children, *n* | 53 | 11,448 |  |
| Female, *n* (%) | 23 (43.4) | 4,968 (43.4) |  |
| Observation period after birth, months, median (range) | 49 (15, 139) | 73 (15, 192) | <0.001 |
| Observation period after birth, categorized |  |  | <0.001 |
| < 1 y, *n* (%) | 0 (0) | 0 (0) |  |
| 1–2 y, *n* (%) | 19 (36) | 1,654 (14) |  |
| 3–4 y, *n* (%) | 11 (21) | 2,631 (23) |  |
| ≥5 y, *n* (%) | 23 (43) | 7,163 (63) |  |
| **Twin delivery** |  |  |  |
| Children, *n* | 12 | 216 |  |
| Female, *n* (%) | 4 (33.3) | 72 (33.3) |  |
| Observation period after birth, months, median (range) | 62 (15, 156) | 79 (16, 178) | 0.507 |
| Observation period after birth, categorized |  |  | 0.096 |
| <1 y, *n* (%) | 0 (0) | 0 (0) |  |
| 1–2 y, *n* (%) | 4 (33) | 24 (11) |  |
| 3–4 y, *n* (%) | 2 (17) | 44 (20) |  |
| ≥5 y, *n* (%) | 6 (50) | 148 (69) |  |

PLO, pregnancy and lactation-associated osteoporosis

^a^Mann-Whitney *U* test and Fisher’s exact test for continuous and categorical variables, respectively
